# A Lateral Flow Device for Point-of-Care Detection of Doxorubicin

**DOI:** 10.3390/bios12100896

**Published:** 2022-10-19

**Authors:** Tania Pomili, Francesca Gatto, Pier Paolo Pompa

**Affiliations:** 1 Nanobiointeractions & Nanodiagnostics, Istituto Italiano di Tecnologia (IIT), Via Morego, 30-16163 Genova, Italy; 2 Department of Chemistry and Industrial Chemistry, University of Genova, Via Dodecaneso, 31-16146 Genova, Italy

**Keywords:** point-of-care, doxorubicin, naked eye, lateral flow device, biological recognition

## Abstract

A simple, rapid, and sensitive point-of-care (POC) device for the on-site detection of doxorubicin was developed. The proposed method relies on the naked-eye detection of the intrinsic fluorescence of the drug in a lateral flow device (LFD) configuration, exploiting the biological recognition of DNA probes and avoiding the use of expensive antibodies and sophisticated instrumentations. The POC assay does not require any pre-treatment or purification step and provides an immediate visual readout, achieving a limit of detection as low as ca. 1 ng doxorubicin, outperforming several laboratory-based instrumental techniques. The POC method was proven useful for the detection of trace amounts of the drug both in the case of water solutions (to simulate surface contaminations) and in urine samples, opening promising perspectives for routine monitoring of doxorubicin, with potential benefit to healthcare workers and personalized chemotherapies.

## 1. Introduction

Doxorubicin belongs to the family of anthracyclines, a class of anticancer agents widely used against a variety of tumors, from breast, ovarian, and thyroid carcinoma to lymphoma and leukemia. The action mechanism of doxorubicin involves its interaction with DNA at different levels: intercalation between the base pairs, strand breakage, and the inhibition of the topoisomerase II enzyme [1]. Although it is frequently used as a first-line anticancer agent, doxorubicin presents many side effects, such as cardiotoxicity, myelosuppression, nausea, and hair loss, which have to be taken into strict consideration during its clinical use [2]. Consequently, its regulated dosage is essential in chemotherapy. Furthermore, the potential toxicity to the exposed healthcare workers in the occupational environment is also emerging as a serious concern [3]. Doxorubicin, indeed, can penetrate protective clothes and, when administered at the therapeutic concentration (2 mg/mL), it was reported to persist on the hand of a volunteer for 30 s, even after washing with common detergents [4]. Therefore, developing affordable methods for the determination of drug contaminations in hospital settings is of great interest.

The analysis of trace amounts of doxorubicin in the clinical departments where the drug is administered to patients is typically performed by laboratory-based instrumental techniques, such as high-performance liquid chromatography, spectroscopic methods, mass spectrometry, and electrochemical sensors [5,6,7,8,9,10,11]. Among these strategies, exploiting doxorubicin intrinsic fluorescence appears a promising and versatile approach. The drug generates strong fluorescence when excited by ultraviolet (~250 nm) or blue light (~465 nm), emitting broad-spectrum orange–red light (530–700 nm range) [12]. Doxorubicin fluorescence properties have been extensively studied in cancer research and imaging [13,14]. However, to the best of our knowledge, only few articles reported the analysis of doxorubicin contaminations on surfaces with this approach [15], especially in the context of point-of-care (POC) sensors. In this framework, a POC device could be interestingly adapted for monitoring drug concentrations in non-invasive biofluids such as urine, improving the workers’ safety as well as the clinical practice, promoting personalized therapies and, hence, reducing the adverse effects [16].

This work aims to develop a simple, rapid, and sensitive POC device for the on-site identification of doxorubicin contamination on surfaces and in urine. The proposed method relies on the naked-eye detection of the drug fluorescence in a lateral flow device (LFD), exploiting the biological recognition of DNA probes, which provides high specificity and sensitivity [17]. This approach also allows avoiding the use of expensive antibodies and sophisticated instrumentations. The LFD was firstly optimized on water solutions to simulate doxorubicin collected from contaminated surfaces and then successfully applied to real urine samples.

## 2. Materials and Methods

### 2.1. Chemicals and Materials

The chemicals employed for this project were of high technical grade and used without further purification. All solutions and buffers were prepared using ultrapure deionized water (MilliQ). Doxorubicin hydrochloride (C_27_H_29_NO_11_···HCl, suitable for fluorescence, 98.0–102.0% (HPLC)), bovine serum albumin (heat shock fraction, protease free, fatty acid free, essentially globulin free, pH 7, ≥98%), Tween-20 (C_58_H_114_O_26_, viscous liquid), sodium dodecyl sulfate (C_12_H_25_O_4_S.Na, BioUltra, for molecular biology, ≥99.0% (GC)), deoxyribonucleic acid from calf thymus (D4522, Type XV, activated, lyophilized powder), poly-D-lysine hydrobromide (P6407, mol wt 70,000–150,000, lyophilized powder, γ-irradiated, BioReagent, suitable for cell culture), chitosan (448,869, low mol wt), Triton™ X-100 solution (laboratory grade, X100), hippuric acid (112,003, 98%), creatinine (C4255, anhydrous, ≥98%), magnesium sulfate heptahydrate (1.05886, Supelco, for analysis EMSURE^®^ ACS, Reag. Ph Eur), sodium sulfate (1.06649, Supelco, anhydrous for analysis EMSURE^®^ ACS, ISO, Reag. Ph Eur), potassium chloride (P9541, for molecular biology, ≥99.0%), sodium chloride (S3014, for molecular biology, DNase, RNase, and protease, none detected, ≥99% (titration)), sodium citrate tribasic dihydrate (C_6_H_5_Na_3_O_7_···2H_2_O, BioUltra, for molecular biology, ≥99.5%), urea (U5378, powder, BioReagent, for molecular biology, suitable for cell culture), uric Acid (U2625, ≥99%, crystalline), ammonia solution 25% (1.05432, Supelco, for analysis EMSURE^®^ ISO, Reag. Ph Eur), sodium phosphate dibasic (71640-M, puriss. p.a., ACS reagent, anhydrous, ≥99.0% (T)), phosphoric acid (695017, ACS reagent, ≥85 wt. % in H_2_O), sodium Hydroxide (221,465, ACS reagent, ≥97.0%, pellets) were purchased from Merck (Sigma-Aldrich). UltraPure™ 1 M Tris-HCI Buffer, pH 7.5 (15,567,027), TE Buffer (20X) (RNase-free, T11493) were purchased from ThermoFisher. For use as a sensing element, 3′ overhangs duplex sequence was designed, and purchased from Integrated DNA Technologies.

Sample pad (grade 319, composition Cotton Fibers), sample pad (grade 8980, composition Chopped Glass w/Binder), and absorbent pad (grade 440, composition Cotton/Glass Blend) were purchased from Ahlstrom-Munksjö. Whatman^®^ qualitative filter paper, Grade 1 (WHA1001090, circles, diam. 90 mm, pack 100), Hybond^®^-N+ hybridization membranes (GERPN203B, W × L 20 cm × 3 m, roll), Amersham™ Protran^®^ Supported Western blotting membranes, nitrocellulose (GE10600016, pore size 0.45 μm, roll W × L 300 mm × 4 m) were purchased from Merck (Sigma-Aldrich). Unisart^®^ nitrocellulose membrane (CN95 backed, 50 m roll) was purchased from Sartorius.

UV lamp (UVP UltraViolet Product™ EL Series UV Lamps, 95-0252-02) was purchased by Fisher Scientific (part of Thermo Fischer Scientific).

### 2.2. Lateral Flow Device Assembly and Electrostatic Immobilization of the Capture Probe

For the realization of the proposed device, the Hybond^®^-N+ hybridization membrane was chosen as the running pad. It was cut to obtain strips of a final dimension of 4 mm × 25 mm, while the absorbent pad was realized with a size of 4 × 18 mm. After the running pad and the adsorbent pad were laminated on the backing card, a cutter was employed to realize strips with a width of 4 mm and a final length of 40 mm, with a 2 mm overlap of the two membranes. For the optimization of the flow, three different running pads (Amersham™ Protran^®^ Supported Western blotting membranes, Unisart^®^ Nitrocellulose Membrane, and Whatman^®^ qualitative filter paper) were tested and their performance was compared with that of the positively charged Nylon membrane. For the immobilization of the capture probe, the electrostatic interaction between the probe and the membrane was exploited. Specifically, 0.5 µL of the 3′ overhangs duplex sequence (200 nmol double-stranded DNA (dsDNA)) were dropped on the test zone, 15 mm far from the beginning of the running pad and in the middle of the strip. Due to the negatively charged phosphate groups of the DNA probe and the positively charged nylon membrane, it was possible to electrostatically immobilize the capture probe on the test zone. After the deposition, the probe was allowed to dry for 60 min at room temperature to ensure dsDNA was completely dried and stably immobilized. For the optimization of the capture probe, solutions of dsDNA with poly-L-lysine and with chitosan were pre-mixed in an equal volume. Specifically, 200 nmol dsDNA were incubated with 0.1 mg/mL aqueous solution of poly-L-lysine hydrobromide and with 0.01% (*m*/*v*) diluted acetic acid solution of chitosan. Thereafter, the solutions were deposited on the test zone and dried at room temperature, as previously described.

### 2.3. Assay Procedure and Data Processing

The proposed device was tested by depositing 30 µL of doxorubicin hydrochloride, previously solved in water and diluted at various concentrations, in 0.1% Triton X-100 solution. Control assays were performed running 30 µL of Triton X-100 solution. In particular, the sample was added at the edge of the running pad, taking approximately 20 s to reach the end of the strip and, hence, the absorbent pad. Considering the wicking capacity of the absorbent pad, 30 µL was established to be the correct volume to avoid overflow and ensure the fluid reaches the end of the strip. For the optimization of the flow, 10 µM doxorubicin was diluted in different solvents, i.e., 0.05% BSA solution in PBS, 0.5% SDS solution in PBS, 100 mM Tris Buffer solution, and 0.1% Tween solution in PBS, and compared with doxorubicin solved in Triton X-100. At the end of the run, the device was analyzed under the UV lamp operating at 254 nm, and a smartphone picture was recorded. A clear fluorescent spot was immediately visualized if doxorubicin amounts were present in the sample. The intensity of the fluorescence was processed through the ImageJ program, recording the green coordinate (G). Doxorubicin has an absorption maximum at 496 nm, which corresponds to the blue/green region of the visible light spectrum. Therefore, G, being the complementary color of the orange–red fluorescence produced in the detection zone, showed the largest change in color values [18,19]. In particular, the images were analyzed through the “color inspector” plug-in of ImageJ, zooming onto the fluorescent spots. ΔG values were obtained by subtracting the G value of the spot obtained when the sample contained doxorubicin from that of the blank (a strip treated with the solvent alone). All the G values represent the average of 9 independent measurements, obtained by processing the relative photos of the devices tested with decreasing doxorubicin amounts. The limit of detection (LoD) of the device was derived from the following equation: (3.3 × Ϭ)/S where Ϭ is the standard deviation of the response and can be determined as the standard deviation of the blanks and S represents the slope of the graph in the linear range [20].

### 2.4. Application in Urine Samples

The proposed device, in the optimized conditions, was firstly tested with synthetic urine, prepared in a laboratory following the protocol of Sarigul et al. [21]. Each component, in the biological concentration, was tested singularly to evaluate the interference with the recognition mechanism. Subsequently, the assay was performed on a urine sample testing the real applicability for routine use. Samples were collected from a healthy volunteer, spiked with the desired doxorubicin concentration, and finally diluted 1:10 in Triton 0.1%. Experiments, both in synthetic and real urine, were carried out in triplicate.

## 3. Results

### 3.1. Device Design and Detection Mechanism

Developing a traditional LFD for the detection of doxorubicin either in direct or in competitive assay format is challenging, due to the very limited commercial availability of anti-doxorubicin antibodies and their remarkably high costs. Therefore, to overcome such issues, we designed and developed an innovative detection strategy, in which the recognition mechanism exploits the pharmacological interaction of doxorubicin with its biological target, namely DNA. Unlike the conventional lateral flow assays, where aptamers or antibodies are commonly employed as capture probes, the design of the proposed LFD here involves the deposition of dsDNA in the test zone as the recognition/capture element. When doxorubicin is present in the test sample, it flows through the device, interacts with the electrostatically immobilized DNA, intercalates in its strands, and remains captured onto the test region. Upon irradiation by a simple UV lamp, the detection zone emits an intense orange/red fluorescent signal, which can be directly detected by the naked eye, with no further instrumentation. Notably, exploiting the pharmacological interaction of the drug with DNA significantly boosts the specificity and sensitivity of the assay.

Nowadays, the development of nucleic-acid-based lateral flow assays (NALFAs) is attracting increasing interest. NALFAs are widely used for the detection of several targets, such as DNA sequences, biomarkers, metal ions, and drugs of abuse [22,23,24,25]. Most of the reported NALFAs are designed with aptamers or single-strand DNAs as sensing probes, and they are commonly used for hybridization-based platforms, where the detection is performed by the complementary recognition of two oligonucleotide strands. For instance, single-strand-based NALFAs have been proposed for the detection of pathogens in food and water [26,27], while aptamers have been reported for the assessment of toxins [28]. However, to the best of our knowledge, no NALFA devices have been developed for the detection of anticancer drugs; moreover, no studies reported the use of dsDNA as recognition/capture probe in the test zone. Herein, we combined such innovative features for the realization of a promising LFD for the specific and high-sensitivity detection of doxorubicin contaminations, exploiting the pharmacological interaction of the drug with the dsDNA, stably immobilized in the test zone.

**Figure 1:**
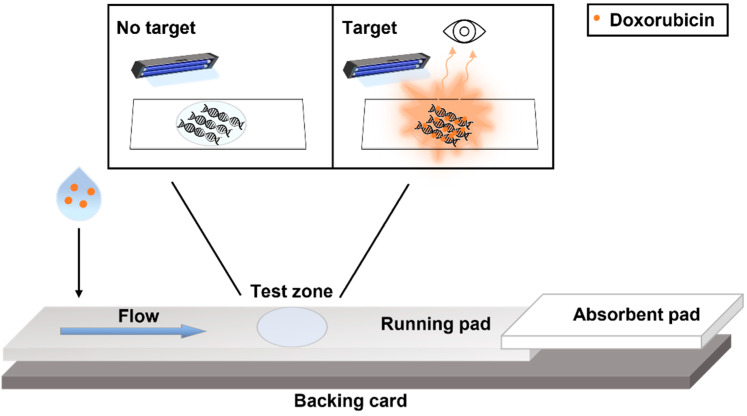
Schematic illustration of the LFD for the assessment of doxorubicin contamination on surfaces and in urine. The device is composed of a running pad made of positively charged nylon and an absorbent pad, both laminated on the backing card and partially overlapped (2 mm). The test zone is realized by dropping 0.5 µL of the DNA solution onto the nylon pad. The test samples are deposited on the lateral side of the running pad and, flowing through the membrane, they reach the recognition element on the test zone. When doxorubicin molecules are present in the test sample, they intercalate with the immobilized DNA probes, giving an intense fluorescence, detectable by the naked eye, when excited by a UV lamp. In the case of non-contaminated samples, no intercalation occurs and, hence, no fluorescence is visualized.

Figure 1 shows the schematic description of the proposed device. In particular, a positively charged nylon membrane was chosen as the running pad, and a blend of cotton and glass fibers was used as the absorbent pad, due to its high wicking capacity. Materials were cut obtaining a width of 4 mm and they were assembled on a backing card for the realization of the device. At a distance of 15 mm from the sample deposition zone, 0.5 µL of dsDNA was placed in the middle of the running pad, acting as the capture probe. Upon deposition of 30 µL of the sample on the running pad (see Section 2, Methods for details), capillary forces enable the analyte to reach the absorbent pad, flowing homogeneously through the membrane and passing across the test zone. When doxorubicin is present in the tested sample, it interacts with the dsDNA probe, and it becomes captured in the test zone. Detection is immediately visualized as an intense fluorescent signal of the test region after irradiation by UV light. In the case of non-contaminated samples, no intercalation occurs and, hence, no fluorescence is visualized.

### 3.2. Optimization of the Capture Probe

Most of the reported techniques for DNA probe immobilization on the running membrane rely on the use of biotinylated capture ssDNA that is pre-complexed with streptavidin. This approach typically allows for efficient probe immobilization along with sensitive detection of target analytes; however, with this system, the costs of the lateral flow devices strongly increase. Other available techniques are physical adsorption and chemical bonding, the latter requiring the use of a linker molecule [29]. In this work, adsorption was selected as the probe immobilization method because of its simplicity and low cost, since it does not require chemical reagents and DNA modification [29,30]. To achieve this goal, four different running pads (nylon+, two different nitrocellulose membranes, and a Whatman filter paper) were tested, comparing their performance after the immobilization of 0.5 µL of DNA on the test zone (see Figure S1, Supplementary Materials). When probed for the capture of a doxorubicin solution, only nylon+ showed a clearly detectable visual fluorescence (under the UV lamp), due to the stability of the DNA probe molecules immobilization in the test zone and the consequent efficient interaction between the drug analyte and the probe. Indeed, the positively charged nylon enables the stable electrostatic adsorption of the negatively charged phosphate group of DNA probes, allowing for the subsequent target detection. On the contrary, the other substrates did not guarantee stable immobilization of the probe molecules in the test region, mainly because probes were washed in the LFD both after the immobilization step and during the test sample flow.

To further increase the ionic interaction between the probe and the surface (to maximize probe stability and its surface density), the literature reports the possibility to functionalize the support creating a positively charged film, e.g., by employing chitosan or poly-L-lysine [29]. We thus tested the performance of our assay, depositing three different t-lines: DNA, poly-L-lysine with DNA, and chitosan with DNA (see Section 2, Methods for details). As it is shown in Figure S2, in our test conditions, no significant advantages were obtained by immobilizing DNA with these agents. Specifically, chitosan led to false-positive results, while poly-L-lysine gave a non-defined signal due to drying process issues.

An additional experimental step was to optimize the DNA sensing probe. In particular, to promote the immobilization process, we selected a short chain (27 nucleotides) duplex oligonucleotide with 3′ overhangs as the capture probe. Overhangs are known to be beneficial to increase the electrostatic interaction between the negatively charged phosphate groups of the nucleotides in the tail and the positively charged surface. Furthermore, smaller oligonucleotides present more flexibility and, hence, favored surface adsorption due to the reduced conformational restrictions [31]. To verify this concept, the assay was tested comparing the performance of the LFD with a t-zone made by DNA from calf thymus, composed of 10 kb pairs, and the selected short sequence. Despite both nucleic acids gave fluorescence in the t-zone upon exposure to UV light, the smaller sequence clearly resulted in a better-defined signal (Figure S3). Calf-thymus DNA was susceptible to the worst drying process, leading to a significant coffee ring effect of the fluorescent spot. Furthermore, we tested the assay upon the addition of decreasing concentrations of doxorubicin, comparing the performance of the two nucleic acids. We observed that the short sequence with the overhangs enabled a higher detection sensitivity along with a homogeneous test spot.

We also evaluated the role of the drying time after the immobilization of the DNA probe in the t-zone. To this purpose, the doxorubicin fluorescence signal was analyzed at four different time points, namely 5, 10, 30, and 60 min (Figure S4). By using short drying times (5 and 10 min), the DNA probe molecules were not completely dried in the t-zone, hence the genetic materials partly followed the flow of the sample till the absorbent pad, producing a strong fluorescent tail. After 30 min, a well-defined signal started to be obtained. However, 60 min gave the best result and, hence, such condition was chosen as the optimal drying time for the assay.

The final optimization of the sensing element concerned the amount of the oligonucleotide immobilized on the t-zone. Specifically, four different DNA concentrations were checked, i.e., 1, 20, 100, and 200 µM. After doxorubicin was deposited on the running pad, we observed that, starting from 100 µM, the fluorescent spot began to be clearly visible and well-defined (Figure S5). However, 200 µM was chosen as the optimal concentration for the assay since it allowed for the best visual response.

### 3.3. Optimization of the Flow

Despite the wide variety of commercially available running pads, which enable a fast and uniform flow, in this work we selected nylon+ for its high binding capacity for nucleic acid probes, as discussed in the previous paragraph. Usually, nylon+ is employed for the Southern blotting technique and, due to its pore size of 0.45 µm, it is not considered a conventional material for the LFD assays, since it allows for a very slow capillary flow rate. Therefore, several tests were performed to promote the samples flowing through the nylon+ running pad uniformly and efficiently. First, sample pads of cotton or glass fibers were tested to assess their suitability for loading the samples, ensuring an even flow [32]. Unfortunately, as shown in Figure S6, both the pads retained a huge amount of doxorubicin, not allowing the drug to properly flow through the running pad and reach the t-zone, thus hampering the detection. Therefore, sample pads were not included in the final design of the device.

We then tried to optimize the sample solvent. When doxorubicin was solved in water, the flow of the drug was uneven and it did not reach the absorbent pad, stopping along the membrane. To allow the analyte to correctly complete the run, we found that two further additions of water were required (Figure S7A). Nevertheless, in this latter case, the running pad resulted not completely clean, with a significant residual fluorescence on the membrane and a front beyond the t-zone. Hence, to improve the flow, doxorubicin was dissolved in several running buffers, and the assay was performed without further additions of solvents. Solutions containing doxorubicin were prepared using BSA, Triton, SDS, Tris Buffer, and Tween (see Section 2, Methods for details). As clearly shown in Figure S7B, only Triton enabled a uniform flow of the drug, allowing its efficient intercalation with DNA probes and providing a good fluorescence signal. All the other tested solvents led to the creation of several fronts on the running pad, hampering the recognition mechanism. Hence, Triton was chosen as the buffering agent in the final device configuration.

### 3.4. Analytical Performance

After all the optimizations discussed above, the performance of the proposed LFD was tested to evaluate the limit of detection (LoD) for doxorubicin contamination.

**Figure 2:**
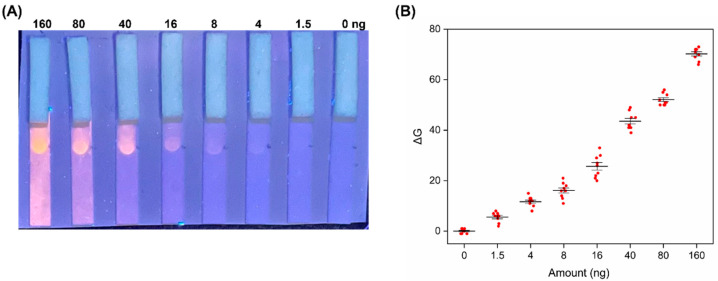
(**A**) Representative photograph of the proposed devices tested with decreasing doxorubicin amounts, ranging from 160 to 0 ng, as reported in the upper part of the image (160, 80, 40, 16, 8, 4, 1.5, 0 ng, roughly corresponding to 10, 5, 2.5, 1, 0.5, 0.25, 0.1 µM); (**B**) analysis of the optical response of the device as a function of doxorubicin amounts (ΔG values were obtained subtracting the G coordinate of the sample from that of the blank (in RGB coordinates system)). G values were recorded in 9 different experiments.

As shown in Figure 2A, 30 µL of decreasing doxorubicin concentrations were tested. As a negative control, 30 µL of triton buffer was also run. Under the UV lamp illumination, a clearly visible spot can be visualized down to a doxorubicin amount of 4 ng. However, a closer inspection reveals that the detection limit appreciable by the naked eye is as low as 1.5 ng doxorubicin. This result has been further confirmed by the image analysis of the fluorescent spots performed on the smartphone photograph (see Section 2, Methods for details of the RGB analysis). The graph reported in Figure 2B shows that there is a good linear relationship between the ΔG values and the doxorubicin amounts over the range tested. As anticipated, the analytical limit of detection of the LFD assay is 1.5 ng (0.1 µM). This means that the POC method is highly sensitive, and the analytical performance is comparable or even superior to other laboratory-based techniques, with the advantage of being rapid and instrument-free (see Table 1).

### 3.5. Detection in Urine

To evaluate the applicability of the proposed method also in the analysis of doxorubicin in urine samples, we performed a series of experiments maintaining the point-of-care concept of our device, namely we did not rely on any purification or pre-treatment of urine samples.

Clinically, the urinary concentration of doxorubicin after the first infusion of the chemotherapeutic drug (0–4 h) was reported to be 95× higher than that observed in plasma. Specifically, Maliszewska et al. showed that the urinary concentration ranges from ca. 17 to 33 µM [38]. As reported in Table 1, most of the reported instrumental techniques have been applied to pharmaceutical formulations due to the possible interactions/interferences between the target analyte and the other components of the biological fluids. Indeed, in many cases, a procedure of pretreatment was required before analyzing the sample and usually the achieved LoD was lower than in aqueous solutions [18]. However, as anticipated, complicated procedures of sample treatment are not suited for a POC device, which is usually applied for near-patient testing without complicated instrumentation.

In this work, we first performed the assay in synthetic urine, in order to understand the possible interference of specific urinary components. In particular, constituents were analyzed singularly running high concentrations of doxorubicin solved in solutions containing each salt at the physiological concentration. As reported in Figure S8, chlorides and ammonia strongly interfere with doxorubicin recognition, hampering DNA bonding. Sodium hydroxide does not completely block the intercalation, but the signal appeared strongly reduced. We could conclude that the interference in our assay conditions arises from a synergic interplay of different constituents rather than a single one. Since it would be challenging to remove such components without resorting to complex pretreatment procedures, we tried to reduce interference effects through a simple pre-dilution step. Interestingly, we observed a partial recovery of the fluorescence signal in the test zone starting from a dilution of 1:5 (Figure S9), while a well-defined and intense spot can be achieved only for higher dilutions (i.e., 1:50). Nevertheless, a good compromise to avoid strong sensitivity loss was represented by the 1:10 dilution.

**Figure 3:**
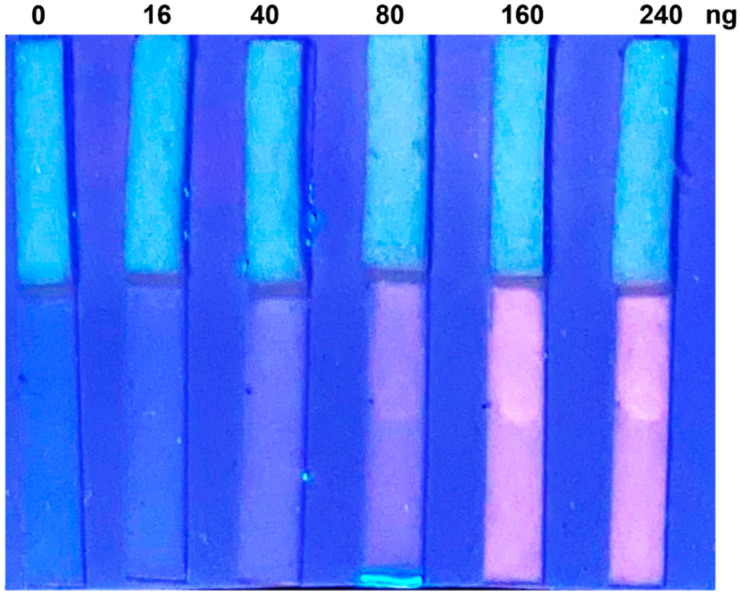
Representative photograph of the LFD devices tested with real urine sample spiked with increasing doxorubicin amounts, as reported in the upper part of the image (240, 160, 80, 40, 16, 0 ng, roughly corresponding to 15, 10, 5, 2.5, 1, 0 µM).

After these preliminary optimizations, we tested the overall performance and sensitivity of the LFD assay running decreasing drug concentrations. A urinary sample from a healthy volunteer was spiked with the target in the 0–250 ng concentration range. Urine was diluted 1:10 in Triton as established by previous experiments, and the fluorescence was recorded under UV lamp. Figure 3 shows the results of the test. As mentioned before, an expected loss in sensitivity with respect to aqueous solution naturally occurred. However, visual detection could be achieved also at a concentration of 80 ng, which is clinically relevant, being much lower than the drug concentration in urine in the first 4 h post-infusion.

## 4. Conclusions

In this work, we showed the development of a portable LFD for the determination of doxorubicin contaminations on surfaces and in urine. The sensing strategy exploits the intrinsic fluorescence of the drug molecule, and the detection approach relies on its capture on the test zone through its intercalation with dsDNA probe, which is the pharmacological target of the drug. Traces of doxorubicin in the test samples (as low as few ng doxorubicin) can be immediately visualized as an intense fluorescence in the test zone, appreciable with a simple UV lamp, with no further instrumentation. The POC device was developed and optimized through a series of systematic experiments. Optimal immobilization of DNA was achieved by exploiting positively charged nylon, which was selected as a running pad because of its electrostatic interaction with the negatively charged phosphate groups of DNA probes. A short dsDNA oligonucleotide with 3′ overhangs was selected as the sensing element since it allowed to further increase its electrostatic adsorption and its surface density, with an overall enhancement of the detection signal. After further optimizations of the LFD device in terms of materials and solvents used, the analytical performance of the device was tested with decreasing concentrations of the target, reaching a visual detection of doxorubicin as low as ca. 1 ng. Remarkably, such naked-eye detection outperforms many instrumental techniques, while being portable, rapid, and not requiring any pre-treatment steps. The LFD device was further tested in a urine sample and also successfully proved its potential applicability for non-invasive drug monitoring in chemotherapeutic patients. Overall, the proposed POC test shows interesting perspectives for routine monitoring of doxorubicin, possibly improving the safety of healthcare workers as well as the quality of clinical practice, paving the way for personalized therapies.

## Figures and Tables

**Table 1:** Comparison of our POC method with other instrumental techniques for the detection of doxorubicin.

Sample Type	LOD (µM)	Preparative Procedure	Visual Detection (Instrument-Free)	Ref.
Pharmaceutical formulations	0.034–0.22	-	X	[33]
Pharmaceutical formulations	0.46	-	X	[34]
Exhaled breath condensate	0.004	Water bath 70 °C for 10 min	X	[35]
Skin and surfaces	1.8	-	✓	[15]
Blood and plasma	0.92	Extraction with ethyl acetate and drying	X	[18]
Cultured cells	0.18	Washing and lysis	X	[36]
Urine samples	0.055	Flow injection	X	[37]
Surfaces and urine samples	0.10	-	✓	This work

## Data Availability

Not applicable.

## Supplementary Materials

The following are available online at https://www.mdpi.com/article/10.3390/bios12100896/s1, Figure S1: Comparison of different running pads, Figure S2: Comparison of different t-lines, Figure S3: Comparison of DNA sensing probes, Figure S4: Optimization of dsDNA drying time, Figure S5: Optimization of dsDNA concentration, Figure S6: Device performance with sample pads, Figure S7: Comparison of different running buffers, Figure S8: Urinary salts interference, Figure S9: Effect of different urine dilutions on the device performance.
