# Supplementary material for: A Lateral Flow Device for Point-of-Care Detection of Doxorubicin
Source: Biosensors (Basel). 2022 Oct 19;12(10):896. doi: 10.3390/bios12100896 (PMC9599782; doi:10.3390/bios12100896)
Supplement: Supplementary file 1 [file biosensors-12-00896-s001.zip › biosensors-1978436-supplementary.pdf]

Supporting Information

A lateral flow device for point-of-care detection of doxorubicin

Tania Pomili [1,2], Francesca Gatto [1], and Pier Paolo Pompa [1,*]

[1] Nanobiointeractions & Nanodiagnostics, Istituto Italiano di Tecnologia (IIT), Via Morego, 30–16163 Genova, Italy
[2] Department of Chemistry and Industrial Chemistry, University of Genova, Via Dodecaneso, 31–16146 Genova, Italy
* Correspondence: pierpaolo.pompa@iit.it

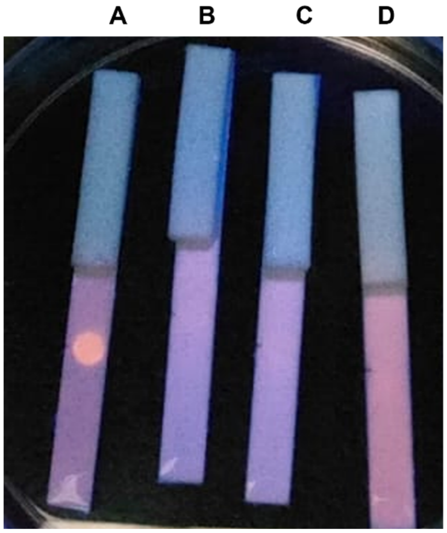

Figure S1. Representative photograph of the devices after running 30 mL of doxorubicin 10 μM, comparing the performance of 4 different running pads: A) nylon+, B) nitrocellulose western blotting membranes, C) nitrocellulose membrane CN95, D) Whatman qualitative filter paper grade 1. Only nylon+ enables the electrostatic adsorption of DNA in the t-line, allowing for the intercalation of doxorubicin and consequent fluorescence emission.

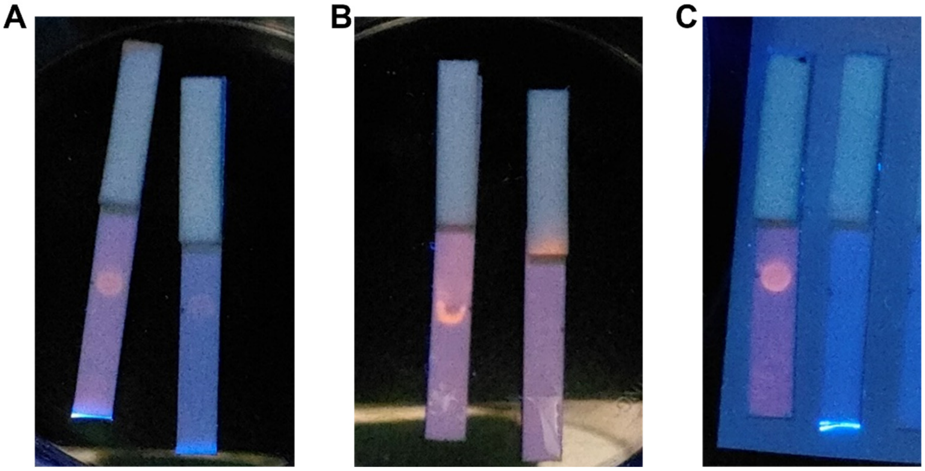

Figure S2. Comparison of the performance of the proposed device after immobilization of A) solution of chitosan and DNA, B) solution of poly-L-lysine and DNA, C) DNA. Devices on the left were treated with a solution of doxorubicin 10 μM, while the ones on the right only with the solvent (negative controls). Chitosan gave false positive results. Poly-L-lysine suffered from uncontrolled drying problems. DNA alone, on the contrary, showed the best signal, with a well-defined spot.

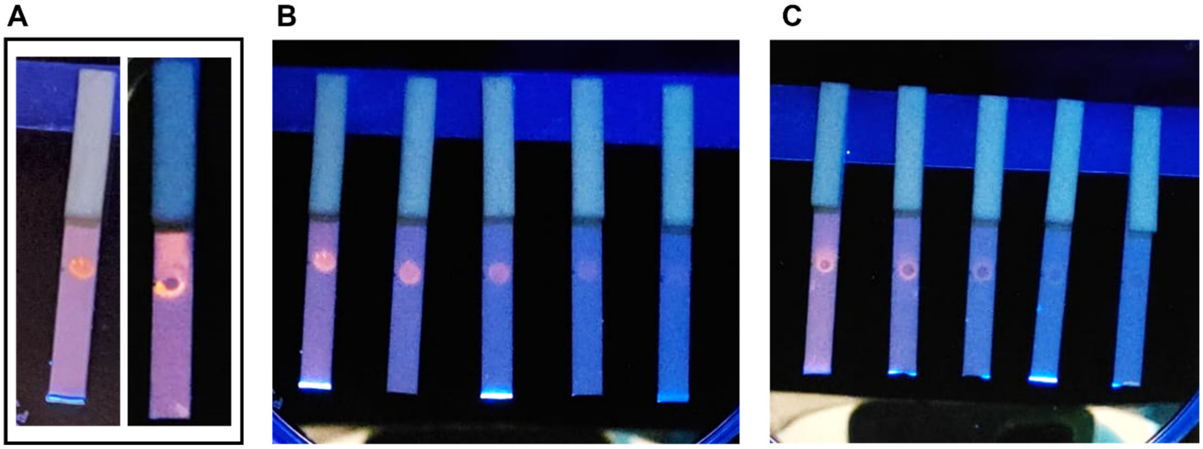

Figure S3. A) Comparison of the performance of the proposed device after immobilization of the dsDNA oligonucleotide with 3′ overhangs (on the left) and DNA from Calf thymus (on the right). B) Photographs of the devices tested with decreasing concentrations of doxorubicin (10, 5, 1, 0.5, 0 μM) after the immobilization of the dsDNA oligonucleotide with 3′ overhangs. C) Photographs of the devices tested with decreasing concentrations of doxorubicin (10, 5, 1, 0.5, 0 μM) after the immobilization of DNA from Calf thymus. DNA from Calf thymus displays drying process problems, leading to a strong coffee ring effect and a worst signal compared with the short oligonucleotide probe.

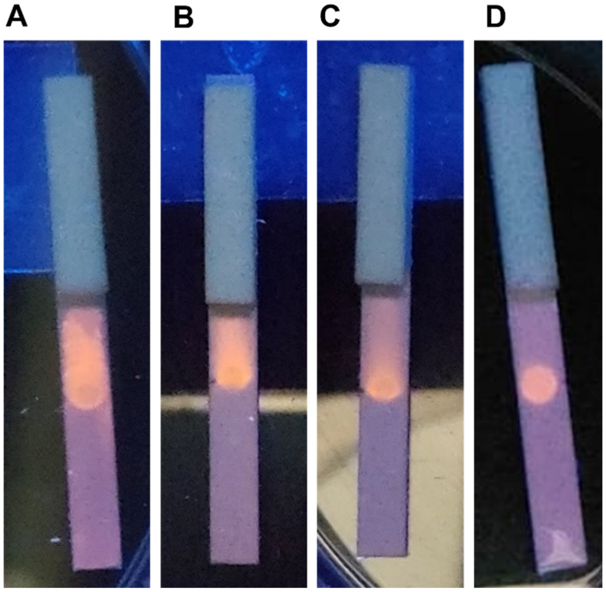

Figure S4. Comparison of different drying times of the test line: A) 5 minutes, B) 10 minutes, C) 30 minutes, and D) 60 minutes. These experiments reveal that 60 minutes is the optimal time required for immobilizing the DNA probe, with a well-defined fluorescent spot and a negligible florescent tail.

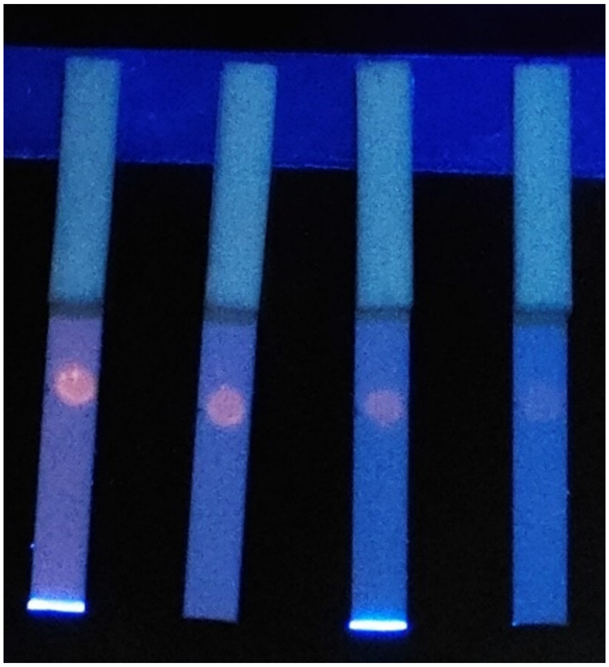

Figure S5. Comparison of the performance of the proposed device after immobilizing different concentrations of the selected oligonucleotide probe. From left to the right: 200, 100, 20, and 10 mM. 200 mM showed the best signal and, hence, it was selected as the optimal concentration.

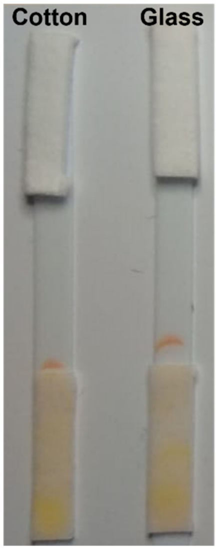

Figure S6. Representative photograph showing the performance of the device after the addition of a sample pad composed of cotton fibers (on the left) or glass fibers. Both pads retain a huge amount of sample.

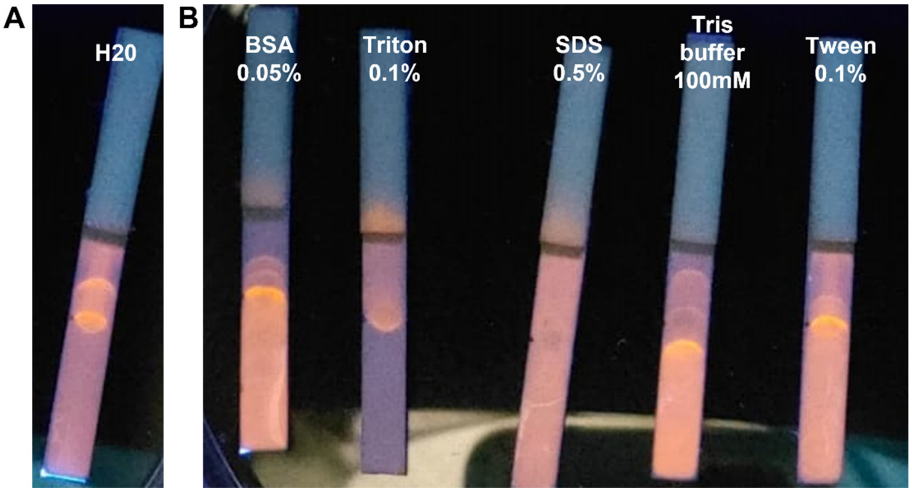

Figure S7. A) Photograph of the device tested with a solution of 10 μM doxorubicin solved in water, with 2 further additions of water to promote the flow. B) Comparison of the performance of the devices after diluting doxorubicin in different running buffers, with no further addition of solvents. Only with triton as running buffer, doxorubicin is allowed to flow smoothly along the membrane.

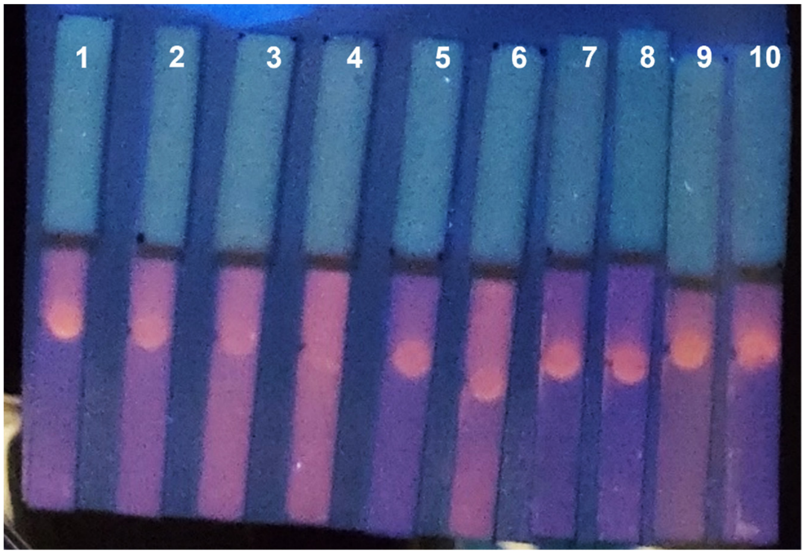

Figure S8. Assessment of the interference of urine salts in the detection of doxorubicin with our proposed LFD. Different solutions of 10 μM doxorubicin were solved in triton buffer (control) (1), a solution of sodium and magnesium sulphates (2), sodium and potassium chlorides (3), ammonia (4), sodium phosphate and phosphoric acid (5), sodium hydroxide (6), sodium citrate (7), urea and uric acid (8), creatinine (9), and hippuric acid (10). The main interferent species are chlorides, ammonia, and sodium hydroxide. Overall, the interference comes from a synergistic interplay of different species rather than a single one.

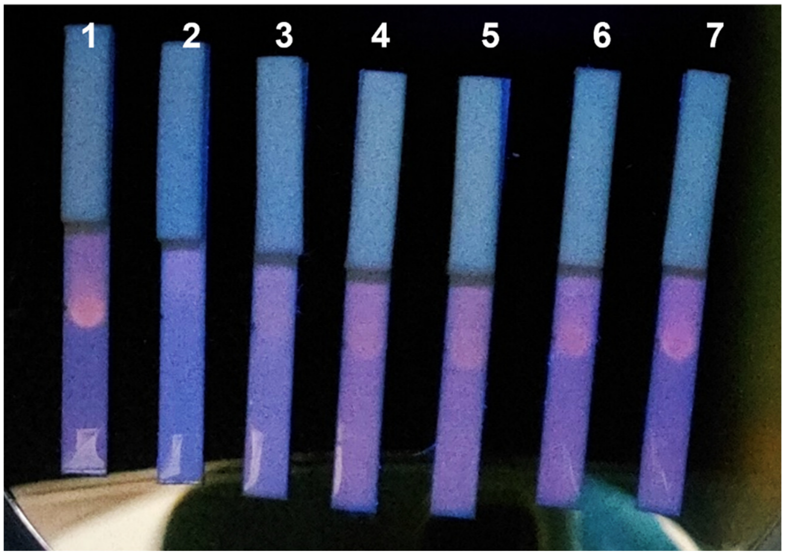

Figure S9. Comparison of the performance of the device after spiking real urine with 10 μM doxorubicin, using several dilutions of the biofluid: no dilution (1), 1:1 (2), 1:2 (3), 1:5 (4), 1:10 (5), 1:25 (6), 1:50 (7). The fluorescent spot starts to be visible with a dilution of 1:5, even if a well-defined signal can be obtained only with 1:50. For avoiding sensitivity loss, a dilution of 1:10 was chosen as the optimized condition.
